# Bet v 1 from Birch Pollen Is a Lipocalin-like Protein Acting as Allergen Only When Devoid of Iron by Promoting Th2 Lymphocytes*

**DOI:** 10.1074/jbc.M114.567875

**Published:** 2014-05-05

**Authors:** Franziska Roth-Walter, Cristina Gomez-Casado, Luis F. Pacios, Nadine Mothes-Luksch, Georg A. Roth, Josef Singer, Araceli Diaz-Perales, Erika Jensen-Jarolim

**Affiliations:** From the ‡ Comparative Medicine Unit, Messerli Research Institute, University of Veterinary Medicine Vienna, Medical University of Vienna, and University of Vienna, A-1210 Vienna, Austria,; the § Biotechnology Department, Center for Plant Biotechnology and Genomics, Technical University of Madrid, 28040 Madrid, Spain,; ¶ AllergyCare, 1220 Vienna, Austria,; the ‖ Department of Anesthesiology, General Intensive Care and Pain Medicine, Medical University of Vienna, Austria, and; the ** Unit of Comparative Immunology and Oncology, Department of Pathophysiology and Allergy Research, Center of Pathophysiology, Infectiology and Immunology, Medical University of Vienna, 1090 Vienna, Austria

**Keywords:** Allergen, Allergy, Immunosuppression, Iron, Mucosal Immunology, Siderophore, Bet v 1, Th2 Skewing, Allergic Sensitization, Apo- and Holo-form

## Abstract

It is hypothesized that allergens are at the borderline of self and non-self and, through as yet elusive circumstances, mount a Th2 response for allergic sensitization. The major birch pollen allergen Bet v 1 is considered the prototype for the PR-10 protein family causing respiratory allergy. Here, we give structural evidence that Bet v 1 is a lipocalin-like protein with a striking structural resemblance to human lipocalin 2. Lipocalin 2 is highly expressed in the lung where it exerts immunoregulatory functions dependent on being loaded with siderophore-bound iron (holo-form) or not (apo-form). We demonstrate that similar to lipocalin 2, Bet v 1 is capable of binding iron via catechol-based siderophores. Thereby, calculated *K_d_* values of 66 nm surpassed affinities to known ligands nearly by a power of 10. Moreover, we give functional evidence of the immunomodulatory capacity of Bet v 1 being dependent on its iron-loaded state. When incubated to human immune cells, only the apo-form of Bet v 1, but not the holo-form, was able to promote Th2 cells secreting IL13. These results provide for the first time a functional understanding on the allergenicity of Bet v 1 and a basis for future allergen immunotherapies counteracting Th2 immune responses on a molecular basis.

## Teaser

**Background:** The reason of allergic sensitization to proteins like Bet v 1 is unknown.

**Results:** Bet v 1 binds iron via catechol-based siderophores. It can modulate human immune cells toward Th2 when not carrying iron.

**Conclusion:** Bet v 1 modulates immune cells toward Th2 when being devoid of iron.

**Significance:** We provide the functional basis under which circumstances Bet v 1 becomes an allergen.

## Introduction

One of the most studied allergens is Bet v 1, the major allergen of birch pollen. Even in classical analyses, it became apparent that among a plethora of proteins in crude birch pollen extract, only a single molecule harbors the IgE binding capacity (1, 2). The greatest majority of birch allergy patients (over >90%) react to Bet v 1, and as a consequence it is used as a marker for birch pollen allergy (3). Bet v 1 was cloned in 1989 and therefore in 2014 celebrates its 25th anniversary (4). To reveal the story behind the exclusive Th2 skewing capacity of Bet v 1, numerous studies subsequently analyzed and are still analyzing its T cell (5, 6) and B cell epitopes (6–8), the impact of its structure (9), or its specific uptake behavior by dendritic cells (10). Ever since structural analyses revealed the hydrophobic pocket within Bet v 1, a rush toward identification of putative ligand has gone on (11–14). Although several ligands have been proposed so far, none of these experiments have functionally explained the actual Th2 skewing capacity of Bet v 1.

Only a few protein families harbor the capacity to generate allergy. In contrast to the plant allergen Bet v 1 being the prototype of the PR-10 protein family (12), most animal-derived allergens belong to the lipocalin protein family (15). Interestingly, lipocalin 2 (LCN2)^4^ is a human homologue to the animal lipocalins. LCN2 is specifically expressed at mucosal barriers where it exerts immunomodulatory functions (16–18) and does so at exactly the same site where respiratory allergens are encountered. Consequently, we hypothesized that a functional relationship might exist that could give some mechanistic insight into allergic sensitization.

## EXPERIMENTAL PROCEDURES

### 

#### 

##### Structural Analysis

**FIGURE 1.**
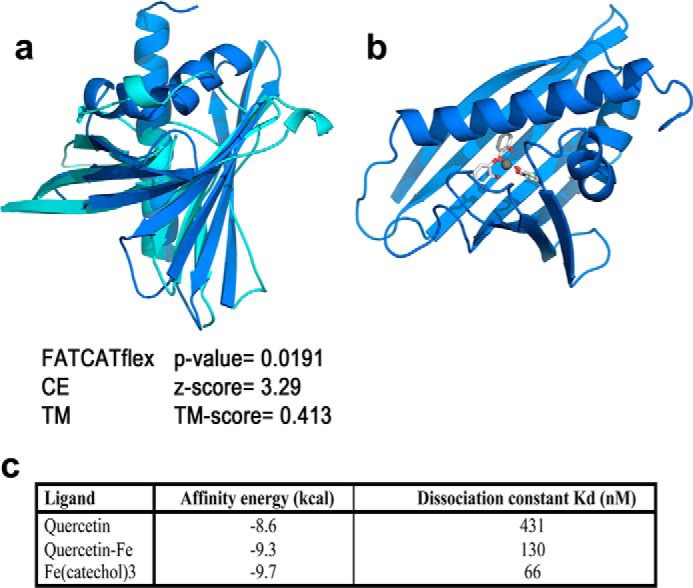
**Structural comparison of Bet v 1 with human LCN 2.** *a*, structural comparison of crystal structures of Bet v 1 (*blue*, 1BV1) and human LCN2 (*cyan*, 1L6M) proteins obtained with FATCATflex. *b*, Fe(catechol)_3_ ligand (*sticks*) docked to Bet v 1 protein.

We employed three different superposition procedures to compare the structure of allergens using (i) the FATCAT (Flexible structure AlignmenT by Chaining Aligned Fragment Pairs) (19), which allows flexibility in structurally aligned blocks by introducing a limited number of twists in the superposed structures; (ii) the CE (Combinatorial Extension) algorithm to align structural fragment pairs (20); and (iii) the TM (Template Modeling)-align procedure, which uses rotation matrices and dynamic programming to optimize a structure alignment (21). Both CE and TM methods yield superpositions referring to the protein structure as a whole, whereas FATCATflex superpositions may give two or more superposed structural blocks if one or more twists are introduced (as it is the case here for Bet v 1: Fig. 1).

For comparisons between crystal structures, FATCATflex and CE superpositions were accomplished by using the j-interface versions of these methods implemented in the PDB Comparison Tool (22). For FATCATflex comparisons involving model structures, the FATCAT server (23) and a version compiled for Linux of the CE program were used instead. TM-align superpositions were carried out in all cases using the TM website (21), which allows comparing experimental as well as theoretical structures. Superposed structures with FATCATflex *p* values < 0.05 are considered significantly similar, CE superpositions with *z*-scores between 3.0 and 4.0 suggest structural similarity, and TM-align superpositions with TM scores between 0.4 and 0.5 indicate significant structural relationship. Structural comparisons between NGAL (neutrophil gelatinase-associated lipocalin) wild-type protein and the allergen Bet v 1 were studied using the x-ray crystal structures (Protein Data Bank (PDB) code in parentheses) for NGAL (1L6M) and birch Bet v 1 (1BV1).

##### Docking Analysis

AutoDock Vina (24) was used to dock the Fe(catechol)_3_ ligand into the hydrophobic pocket of Bet v 1. The protein was taken from the crystal structure of the Bet v 1-naringenin complex, PDB code 4A87 (13). The structure of the ligand was taken from our previous study on the binding of iron-catecholates to lipocalins (25). Docking input files for Bet v 1 and Fe(catechol)_3_ were then generated with AutoDock Tools (26). A grid box of 20 × 24 × 28 Å with origin at the center of the hydrophobic pocket of Bet v 1 was used in AutoDock Vina calculations. The docking geometry with lower affinity energy (−9.7 kcal/mol) was selected to model the Bet v 1-Fe(catechol)_3_ complex (Table 1).

**TABLE 1:** **Affinity energy and dissociation constant of siderophores**

Ligand	Affinity energy	Dissociation constant *K_d_*
	*kcal*	*nm*
Quercetin	−8.6	431
Quercetin-iron	−9.3	130
Fe(catechol)_3_	−9.7	66

##### Generation of Holo-Bet v 1

Recombinant Bet v 1a was incubated with an equimolar concentration of iron (ammonium iron (III) citrate, Sigma) and a 3-fold molar concentration of catechol because three catechols are necessary for hexadental binding of iron in the LCN2 pocket against deionized water.

##### Isolation of PBMCs

The study was approved by the Institutional Ethics Committee of the Medical University of Vienna and conducted in accordance with the Helsinki Declaration of 1975. Ten volunteers donated 15 ml of blood. All subjects gave their full written informed consent.

Blood was mixed with equal volumes of PBS containing 2% FCS before applying onto 10 ml of Ficoll-Paque PLUS (GE Healthcare, Uppsala, Sweden) followed by centrifugation at 400 × *g* for 30 min without break and washing the cells twice with 0.89% sodium chloride solution. Cells were then diluted to a concentration of 1 × 10^6^ cells/ml in RPMI medium containing 10% FCS. Throughout the study FCS from the same lot was used.

##### Stimulation of PBMCs

To peripheral blood mononuclear cells (PBMCs) (0.5 Mio/test), a final concentration of 0.75 ng/ml phorbol 12-myristate 13-acetate (PMA), 100 μg/ml apo*-*Bet v 1 in the presence or absence of 30 μm catechol (Sigma) and 10 μm iron was added. Controls included PMA alone or in the presence of 30 μm catechol (Sigma) and 10 μm iron. PMA concentration was determined in pre-experiments and considered optimal when cells were slightly down-regulating surface CD4^+^ expression (27). After 18 h supernatants were collected and stored at −80 °C until further analysis.

Cells were stained for 30 min at 4 °C with CD3-APC (clone SK7, eBioscience (Santa Clara, CA)), CD4-PE-Cy7 (clone SK3, BD Biosciences), and CD8-PE (clone SK1, BD Biosciences), in PBS containing 2% FCS followed by a 10-min incubation of annexin V FITC (BD Bioscience) and 7-amino-actinomycin D (eBioscience) in binding buffer (10 mm Hepes, 140 mm NaCl, 2.5 mm CaCl_2_) at room temperature. Acquisition and analysis were performed on a FACSCanto II machine (BD Biosciences) using the FACSDiva Software 6.0.

##### Determination of Cytokines

IL13 and IFNγ were detected with commercially available kits according to the manufacturers' protocol using undiluted supernatants. ELISAs for human IL13 and IFNγ were from eBioscience, both assays having a reported sensitivity of 4 pg/ml.

##### Statistical Analysis

Statistical analyses were conducted with repeated measures analysis of variance following Newman-Keuls multiple comparison test using GraphPad Prism 5 software (GraphPad, San Diego, CA). *p* < 0.05 was considered statistically significant.

## RESULTS

### 

#### 

##### Bet v 1 Resembles Human Lipocalin 2

In our first approach, we compared the structure of the major birch pollen allergen Bet v 1 with human LCN2 using three different superposition procedures (FATCATflex, CE, and TM). Superimposition of Bet v 1 (1BV1) with LCN2 (1L6M) revealed a very similar core structure around the hydrophobic pocket (*p* value 0.0126, score 126.12, root mean square deviation 3.15). Further analysis with CE superpositions resulted in a *z*-score of 3.29, suggesting structural similarity, and TM-align superpositions calculated a TM score of 0.413, indicating significant structural relationship.

##### Affinity of Bet v 1 to Iron-Siderophore Complexes

For docking experiments, the crystal structure of the Bet v 1-naringenin complex was taken from PDB code 4A87 (13), and the structure of the ligand was taken from our previous study on the binding of iron-catecholates to lipocalins (25). Docking analyses with the previously reported natural ligand quercetin resulted in affinity energy of −8.6 kcal/mol and a dissociation constant, *K_d_*, of 431 nm. The virtual addition of Fe(III) to the quercetin ligand improved affinity energy to −9.3 kcal/mol and lowered *K_d_* to 130 nm. Binding of iron-catecholates to Bet v 1 resulted in −9.6 kcal/mol affinity energy and a *K_d_* of 66 nm. Hence, the addition of iron improved affinity energy greatly, suggesting that Bet v 1 might function as an iron carrier protein.

##### Apo- but Not Holo-Bet v 1 Promotes Th2 Cells

**FIGURE 2.**
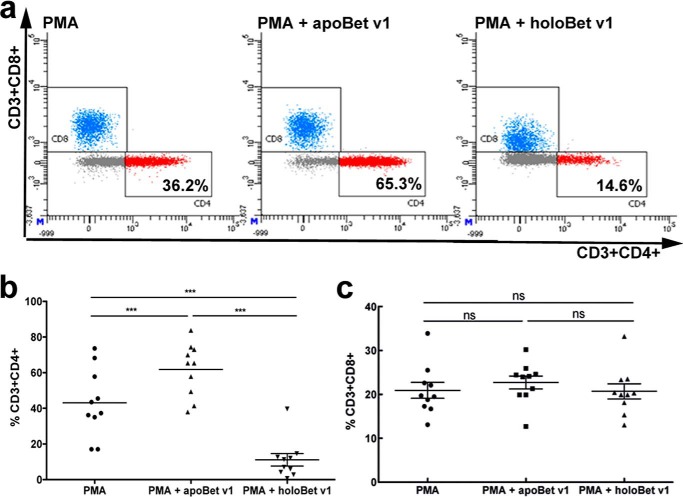
**Apo-, but not holo-Bet v 1 promotes Th2 cells.** PBMCs were treated with PMA alone or in the presence of apo- or holo-Bet v 1. *a*, representative pictograms of CD3 gated PBMCs plotted for CD4 and CD8. *b*, percentage of CD3^+^CD4^+^ cells. *c*, percentage of CD3^+^CD8^+^ cells. Statistical analyses were conducted with repeated measures analysis of variance following Newman-Keuls multiple comparison test. ***, *p* < 0.001, *ns*, not significant.

**FIGURE 3.**
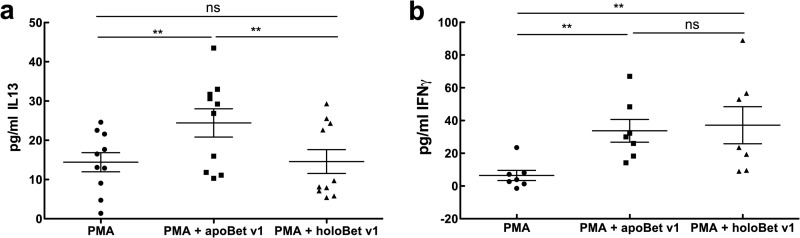
**Apo-, but not holo-lipocalin allergen promotes secretion of IL13.** *a* and *b*, IL13 levels (*a*) and IFNγ levels (*b*) of stimulated PBMCs. Statistical analyses were conducted with repeated measures analysis of variance following Newman-Keuls multiple comparison test. **, *p* < 0.01, *ns*, not significant.

**FIGURE 4.**
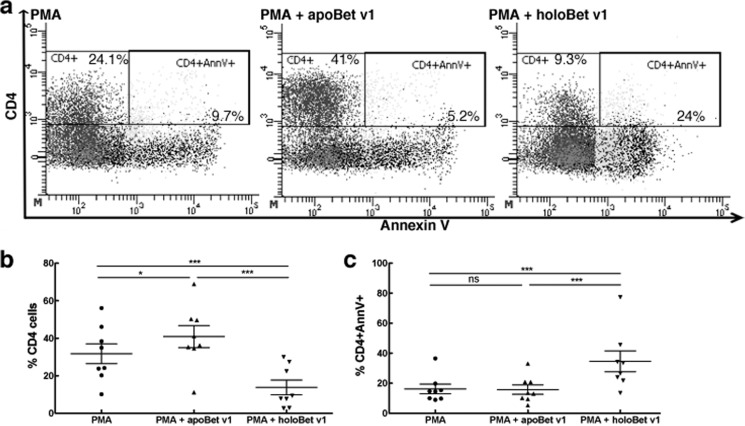
**Annexin V staining of CD4 cells.** PBMCs were treated with PMA alone or in the presence of apo- or holo-Bet v 1. *a*, representative pictograms of PBMCs plotted for CD4 and annexin V (*AnnV*). *b*, percentage of CD4 cells. *c*, percentage of annexin V-positive CD4 cells. Statistical analyses were conducted with repeated measures analysis of variance following Newman-Keuls multiple comparison test. ***, *p* < 0.001, *, *p* < 0.05, *ns*, not significant.

LCN2 is able to drive immune responses dependent on its iron-loading state. Consequently, we next tested whether the holo- or apo-birch pollen allergen Bet v 1 was also able to skew the immune system toward Th2, thereby enabling isotype switch and the generation of IgE antibodies (18, 19). We activated PBMCs from healthy and allergic individuals with a low concentration of PMA, and apo- or holo-Bet v 1 for 18 h *in vitro*, and analyzed CD3 cells for their CD4 and CD8 expression. Further, we checked the secretion of typical Th1 (IFNγ) and Th2 (IL13) cytokines. Indeed, Bet v 1 stimulation was associated with an increased percentage of CD3^+^CD4^+^ lymphocytes (Fig. 2, *a* and *b*), but only when the apo-form was introduced. CD8^+^ numbers were not affected when applying lipocalin allergens in either the apo-form or the holo-form to immune cells (Fig. 2, *a* and *c*). The promoted CD4^+^ cells could indeed be classified as Th2-cells because IL13 could already be detected after 18 h in their supernatants (Fig. 3). No differences in IFNγ secretion between apo- or holo-Bet v 1 were observed (Fig. 3). In contrast, holo-Bet v 1 significantly reduced the numbers of CD3^+^CD4^+^ cells, but not of CD8^+^ cells. Additionally, no IL13 was produced (Figs. 2 and 3). CD4^+^ cell expression was noticeably up-regulated upon the addition of apo-Bet v 1, whereas the addition of holo-Bet v 1 drastically reduced CD4^+^ expression. A significantly higher percentage of CD4^+^ cells were positive for annexin V when cells were incubated with holo-Bet v 1, which was not observed when cells were incubated with apo-Betv1. Hence, relatively more CD4 cells underwent apoptosis when human immune cells were exposed to holo-Bet v 1 (Fig. 4). Thus, Bet v 1 was able to mount a Th2 response in its apo-form, but it abrogated an immune response partly by induction of apoptosis when introduced to immune cells in its holo-form.

## DISCUSSION

The mechanisms of sensitization are poorly understood, and most data have focused on allergic reactions of the respiratory tract rather than on the initial steps of allergic sensitization. We hypothesized that the nature of allergens *per se* contributes to the priming process and that priming occurs when allergens interfere with our own immune system.

Only a very limited number of protein families are truly able to cause allergic sensitization. Allergens predominantly causing respiratory allergies usually belong to the lipocalin protein family comprising nearly all major animal-derived allergens (15), and plant major allergens usually originate from the prolamin or cupin superfamilies, as well as the family so far being classified as PR-10 (12).

One interesting aspect of these proteins, which is usually not addressed, is that despite their high allergenic potential, they usually have a very low immunogenicity (28). They seem to suppress the immune system rather than to activate it. Considering that most people tolerate them fairly well, this should be the normal situation. However, under certain circumstances, this normal immune response is skewed.

To our surprise, the major birch pollen allergen Bet v 1 had a striking structural resemblance to human LCN2 when we compared it with human LCN2. The resemblance of Bet v 1 to LCN2 is remarkable considering its distant phylogenetic relation and prompted us to the hypothesis that the biological function of Bet v 1 might be similar to LCN2 as well.

Recently, the natural ligand of Bet v 1 was identified as being quercetin (11), which is a catechol derivate known for its ability to bind iron (29). Consequently, we next calculated *in silico* the affinity of Bet v 1 to quercetin alone, in conjunction with iron, or to Fe(catechol)_3_ by docking analysis (24). Confirming our hypothesis, affinity was increased 3-fold when iron was added to the natural ligand quercetin, and binding affinity of iron complexed with three catechols, Fe(catechol)_3_, even reached a *K_d_* value of 66 nm. The binding to iron-siderophore thereby was calculated as being nearly 10-fold stronger than with any other ligand described so far.

Our data using Bet v 1 as a model allergen clearly demonstrate that the ligand is decisive in modulating the immunosuppressive environment toward Th2. We provide here for the first time data suggesting Bet v 1 as a carrier for iron-siderophore complexes.

Moreover, we give evidence that the mode of action by which Bet v 1 is able to skew the immune system toward Th2 is similar to that seen by the immunoregulatory protein lipocalin 2, the function of which depends on its iron-loaded state. We show that Bet v 1 *per se* was able to mount a Th2 response when the allergen was introduced to the immune cells in the apo-form, and we give evidence that applying the protein in its holo-form can inhibit the Th2-skewing potential of Bet v 1.

In this study, we focus our attention on the initial steps of allergic sensitization. Here, we definitely could demonstrate that the apo-form of Bet v 1 is able to prime T cells toward Th2. We did not yet address whether the induced T cells are also important in the effector phase, when re-exposure to the allergen occurs. We anticipate that because allergic individuals, once sensitized, remain allergic to birch, the holo-form of Bet v 1 is also capable of eliciting allergic reactions.

We believe that the obtained results have significant implications not only for the understanding of the origin of an allergen-induced Th2 response, but likely for improvement of allergen immunotherapy as well. In light of this knowledge, we consider Bet v 1 to be a lipocalin allergen, contributing thereby further to a shrinking of the list of protein families known to cause sensitization.

Hence, we postulate that under steady-state conditions, allergens are immunosuppressive when presented in the holo-form to the immune system. We hypothesize that under certain conditions such as inflammation or infections, in which immune cells are iron-deprived, allergens lose their iron-siderophore complexes and are presented to the immune system in their apo-form, leading to a Th2 skewing.

The efficacy of allergen immunotherapy up to now (30) has varied dependent on the allergen extracts used, in particular because the exact mechanisms of desensitization are a complex interplay of immunosuppressive factors. The intrinsic potential of natural Bet v 1 to trigger IgE-mediated responses, undesired during allergen immunotherapy, has been understood and circumvented by the construction of many hypoallergenic variants of Bet v 1, which have entered clinical trials (31). Allergen immunotherapy is still a long-lasting treatment, finally leading to the desired Th1 shift (32). In light of the current findings, we are prompted to state that in the future, spiking of apo-Bet v 1 with iron will be a simpler approach and will likely improve the efficacy of allergen immunotherapy against birch pollen.

## Footnotes

4 The abbreviations used are:

LCN2: lipocalin 2
FATCAT: Flexible structure AlignmenT by Chaining Aligned Fragment Pairs
CE: Combinatorial Extension
TM: Template Modeling
PBMC: peripheral blood mononuclear cell
PMA: phorbol 12-myristate 13-acetate.
